# Cross-Correlations between Scientific Physical Fitness, Body Mass Index Distribution, and Overweight/Obesity Risks among Adults in Taiwan

**DOI:** 10.3390/medicina58121739

**Published:** 2022-11-27

**Authors:** Chang-Tsen Hung, Po-Fu Lee, Chi-Fang Lin, Chien-Chang Ho, Hui-Ling Chen, Jenn-Woei Hsieh, I-Tung Lin, Hsing-Chun Kuo, Yu-Ting Lin, Yun-Tsung Chen

**Affiliations:** 1 Department of Health and Leisure Management, Yuanpei University of Medical Technology, Hsinchu City 306, Taiwan; 2 Department of Leisure Industry and Health Promotion, National Ilan University, Yilan County 260, Taiwan; 3 College of Humanities and Management, National Ilan University, Yilan County 260, Taiwan; 4 Exercise and Recreation Development Center, National Ilan University, Yilan County 260, Taiwan; 5 Department of Physical Education and Sport Sciences, National Taiwan Normal University, Taipei City 106, Taiwan; 6 Department of Physical Education, Fu Jen Catholic University, New Taipei City 24205, Taiwan; 7 Research and Development Center for Physical Education, Health and Information Technology, College of Education, Fu Jen Catholic University, New Taipei City 24205, Taiwan; 8 Sports Medicine Center, Fu Jen Catholic University Hospital, New Taipei City 243, Taiwan; 9 Graduate Institute of Educational Leadership and Development, Fu Jen Catholic University, New Taipei City 242, Taiwan; 10 Office of Physical Education, Fu Jen Catholic University, New Taipei City 24205, Taiwan; 11 College of General Education, Chihlee University of Technology, New Taipei City 220, Taiwan; 12 Graduate Institute of Sport Coaching Science, College of Kinesiology and Health, Chinese Culture University, Taipei City 111, Taiwan

**Keywords:** physical fitness, body composition, BMI, adiposity, 3MPKS

## Abstract

*Background and Objectives*: Health-related physical fitness reduces the risk of chronic disease, promotes quality of life, and has enormous economic benefits considering the global health care costs resulting from obesity. However, relatively limited information is available regarding the dose–response relationship between scientific physical fitness and obesity risk. This study aimed to determine the associations of scientific physical fitness with body mass index (BMI) distribution and overweight/obesity risk among adults aged 23–64 years in Taiwan. *Materials and Methods*: We conducted a cross-sectional study and reviewed data derived from the Scientific Physical Fitness Testing Program, Sports Administration, Ministry of Education, Taiwan. Responses from 16,939 participants from the database (7761 men and 9178 women, aged 23–64 years) were collected in this study. Each participant completed a series of scientific physical fitness measurements, including cardiorespiratory fitness (3 min progressive knee-up and step [3MPKS] test), muscular fitness (hand grip strength), and flexibility (sit-and-reach test). Anthropometric measurements included body height, weight, and BMI. The quartiles of scientific physical fitness results were identified as the dependent variable in the multiple linear and multiple logistic regression analysis to determine the associations of the scientific physical fitness measurements with BMI distribution and overweight/obesity risk, as well as the dose–response relationship. *Results*: The 3MPKS test was significantly associated with BMI (quartile 1 (Q1): *β* = 1.900; quartile 2 (Q2): *β* = 1.594; quartile 3 (Q3): *β* = 1.079 for men, and Q1: *β* = 1.454; Q2: *β* = 0.882; Q3: *β* = 0.555 for women), overweight (Q1: odds ratio (OR) = 2.117; Q2: OR = 2.056; Q3: OR = 2.063 for men, and Q1: OR = 3.036; Q2: OR = 2.542; Q3: OR = 1.959 for women), and obesity (Q1: OR = 6.530; Q2: OR = 5.747; Q3: OR = 3.557 for men, and Q1: OR = 3.238; Q2: OR = 1.431 for women) risk compared with quartile 4 (Q4) as the reference group with a dose–response relationship. In addition, relative hand grip strength was significantly associated with BMI (Q2: *β* = −0.922; Q3: *β* = −1.865; Q4: *β* = −3.108 for men, and Q2: *β* = −1.309; Q3: *β* = −2.161; Q4: *β* = −2.759 for women), overweight (Q2: OR = 0.806; Q3: OR = 0.697; Q4: OR = 0.278 for men, and Q2: OR = 0.667; Q3: OR = 0.398; Q4: OR = 0.228 for women), and obesity (Q1: OR = 0.528; Q2: OR = 0.206; Q3: OR = 0.049 for men, and Q1: OR = 0.351; Q2: OR = 0.129; Q3: OR = 0.051 for women) risk compared with Q1 as the reference group with a dose–response relationship. *Conclusions*: Higher levels of performance of the 3MPKS and relative grip strength tests were associated with lower BMI and overweight/obesity risk in both sexes. However, the sit-and-reach test was only partially related to BMI and overweight/obesity risk in both sexes. Cardiorespiratory fitness and muscular fitness were effective predictors of BMI distribution and overweight/obesity risk in Taiwanese adults.

## 1. Introduction

The World Health Organization (WHO) recently reported that more than 1.9 billion adults worldwide were overweight, and at least 650 million of them were classified as obese [1]. Obesity has reached the global epidemic dimension, and is associated with an increased risk of cardiovascular disease (CVD), hypertension, diabetes, osteoarthritis, and cancers, thereby reducing quality of life and increasing the risk of premature death [2,3]. Notably, 44% of Taiwanese adults were recently classified as overweight and obese [3]. Therefore, the successful prediction of future risk for overweight and obesity and subsequent weight management are important topics in Taiwan.

Body mass index (BMI) is commonly used to classify the obese status in adults. The WHO suggested cutoff points for overweight and obesity of greater than 25 kg/m^2^ and 30 kg/m^2^, respectively [1]. Studies have found that BMI is a good predictor of multiple health outcomes, such as heart disease, diabetes, osteoarthritis and anxiety [4,5]. In addition, health-related physical fitness, including cardiorespiratory fitness, muscle strength and endurance, flexibility, and body composition, is associated with health [6]. It has been shown that higher levels of cardiovascular fitness and muscle strength are associated with lower risks of CVD, metabolic syndrome (MS), stroke, and osteoporosis [7]. Flexibility has positive effects on the range of motion of the joints, body stability, and relaxation, as well reducing sports injury and MS risks [8,9,10].

The standard measurement of cardiorespiratory fitness is maximal oxygen uptake (VO2max), which may be obtained from direct measurements or indirect estimates [6]. There was a significant negative correlation between VO2max and BMI in young adults [11]. Compared with direct testing in a laboratory setting, the 3 min progressive knee-up and step (3MPKS) test is a more time-efficient and valid method for predicting VO2max as it does not require expensive metabolic equipment or the need to exercise to the point of exhaustion [12]. In addition, grip strength is a good predictor of overall body strength [13] and has been shown to be associated with physical performance, cardiometabolic health, and quality of life [14,15]. It has been reported that relative grip strength (e.g., kilograms divided by body weight or BMI) was more strongly associated with CVD and MS risks than absolute grip strength [16,17]. A British cohort study showed that higher grip strength was associated with higher BMI [18]. However, higher grip strength is associated with lower waist circumference (WC; an indicator of central adiposity), after adjustment for BMI. A similar study indicated that relative grip strength was negatively associated with WC in young adults [19], and the use of properly adjusted confounding factors (e.g., age, education, occupation, and physical fitness) was necessary to prevent bias and distortion [20].

Health-related physical fitness reduces the risk of chronic disease, promotes quality of life and has enormous economic benefits considering the global health care costs resulting from obesity [21]. However, relatively limited information is available regarding the dose–response relationship between scientific physical fitness and obesity risk. To provide obesity prevention strategies, detailed knowledge of scientific physical fitness levels in the Taiwanese population is required. Therefore, our study aimed to assess the 3MPKS test performance, relative hand grip strength, and sit-and-reach of the Taiwanese population and their impact on BMI distribution and overweight/obesity risk.

## 2. Materials and Methods

### 2.1. Study Design and Participants

This cross-sectional study is based on deidentified data from Taiwan’s Scientific Physical Fitness Testing Program (TSPFTP). The TSPFTP was conducted by the Sports Administration of the Ministry of Education in Taiwan, to obtain annual data from scientific physical fitness tests performed by Taiwanese adults aged 23 to 64 years. The design of this survey used convenience sampling. Participants were voluntarily recruited through the Internet from 18 physical fitness test stations in Taiwan. The survey included face-to-face interviews followed by a standardized structural questionnaire, anthropometric measurements, and scientific physical fitness tests conducted by trained examiners and medical specialists (usually nurses or doctors). This study was conducted in accordance with the Declaration of Helsinki, and the protocol was approved by the Institutional Review Board of Fu Jen Catholic University in Taiwan (FJU-IRB C110113).

### 2.2. Data Collection

Data collection took place from September to November 2017 and included three different approaches. First, trained examiners and medical specialists checked the resting heart rate (HR), and systolic and diastolic blood pressure of the participants, as well as assessing their potential safety risks using the Physical Activity Readiness Questionnaire (PAR-Q). After 15–20 min of rest, the resting HR and brachial systolic blood pressure were measured using telemetry (Polar RS800CX; Polar Electro Oy, Kempele, Finland) and an automatic blood pressure monitor (HEM-7000-C1; OMRON Healthcare Co., Ltd., Kyoto, Japan), respectively. Systolic blood pressure was taken twice, and if the values were not within 5 mm Hg, a third measurement was conducted [22]. The main eligibility criteria were that participants were male or female (≥18 years old) and lived in Taiwan. Exclusion criteria included: (1) participants who were hypertensive (≥130/80 mmHg); (2) participants who had any known cardiorespiratory disease or lower extremity muscle injuries that might hinder them from performing the 3MPKS test. A total of 18,691 participants passed the preliminary safety assessment and were then allowed to proceed to the next step.

Second, participants were requested to complete the demographic questionnaire (or verbally answer the questions), as well as to complete the anthropometric measurements. After completing the questionnaire, the participants were instructed to warm up (dynamic and static muscle stretching) for approximately 10 min. Then, a series of physical fitness measurements were performed, with interval break permitted for 2–4 min between measurements. For this study, a total of 16,939 participants (7761 men and 9178 women) who passed the potential safety risk assessment, completed the questionnaire and underwent the physical fitness measures were finally enrolled in the analysis.

### 2.3. Demographic Characteristics

Through face-to-face interviews, a standardized, structured questionnaire was used to collect the data on demographic characteristics (i.e., age and gender), socioeconomic status (i.e., education, monthly income, marital status, and relationship status), and the residence zip code. Education was divided into elementary school or lower, junior or senior school, and college or higher. Currently employment status was divided into yes, no, and other. Monthly income was divided into ≤ 20,000 NTD (new Taiwan dollar), 20,001–40,000 NTD, and ≥ 40,001 NTD. Marital status was divided into married, never married, and divorced/separated/widowed. Relationship status was divided into living with someone and not living with someone.

### 2.4. Anthropometric Measures

The anthropometric measurements included body height, weight, and BMI, taken after the participants were asked to remove their shoes and heavy clothes and stand in a normal posture. Body weight and height were measured in meters to the nearest 0.1 kg and 0.1 cm, respectively, using an electronic height–weight scale. In addition, BMI was calculated based on body weight and height (weight (kg)/height (m)^2^), and the BMI categories of normal weight, overweight, and obesity were defined as a BMI of 18.5 ≤ BMI < 24 kg/m^2^, 24 ≤ BMI < 27 kg/m^2^, and BMI ≥ 27 kg/m^2^, respectively, in accordance with the Health Promotion Administration in Taiwan [23].

### 2.5. Scientific Physical Fitness Measurements

After the anthropometric measurements, the following scientific physical fitness tests were conducted: cardiorespiratory fitness was measured via the 3MPKS test (mL/kg/min) [12], muscular fitness was measured via hand grip strength (kg) [24], and flexibility was measured using the sit-and-reach test (cm) [25]. All the participants performed the tests in the following order with a sufficient break period (3–5 min) between tests: hand grip strength, sit-and-reach test, and 3MPKS test. Participants were asked to avoid any other vigorous- or moderate-intensity physical activity before performing these tests. The examiner introduced a 10 min warm-up which the participant completed before the scientific physical fitness assessment.

The 3MPKS test was measured using an HR monitor worn by each participant during the testing process. After putting on the HR monitor, the participants stood while the midway point between their patella and iliac crest was measured as the target height for lifting the knees and was marked by colored tape. Once the test had been initiated, the participants were asked to match a rhythm produced by an electronic metronome while stepping on the spot and raising their knee to the marked height with each step. The 3MPKS test started at 96 steps per minute (SPM), and the rate was increased by 24 SPM every minute. If the participants were unable to maintain the rhythm, they could run instead of walk for up to 3 min. If the participants were unable to lift the knees to the required height or if they could not follow the rhythm for 30 s, then the test session was terminated and the results were eliminated from the analysis. For safety reasons, participants had to maintain a step rate of 80 SPM for a cool-down period of 30 s before resting in a standing position. The HR recorded data at the beginning of exercise testing (HR0); at the first (HR1), second (HR2), and third minutes (HR3) during the exercise testing; and at the first minute postexercise testing (HR4) [12]. The predicted VO2max was calculated as follows [12]: 72.334 − 0.261 × age + 4.366 × gender (1 = men, 0 = women) − 0.448 × fat% − 0.134 × HR0 − 0.082 × (∆HR3 − HR0) + 0.073 × (∆HR3 − HR4).

Hand grip strength was measured using an electronic hand grip dynamometer by taking the average of the two handgrip attempts. The participants stood up with their hands hanging down naturally, and held the grip dynamometer with their dominant hand. The averaged value of the hand grip strength was then divided by body weight, and the relative grip strength was recorded for analysis.

Participants were asked to perform the sit-and-reach test twice using a sit-and-reach box with a measuring scale, where 30 cm was at the level of the feet, and the average distance from the two attempts was used for analysis.

### 2.6. Statistical Analyses

All statistical analyses were performed using SAS version 9.4 software (SAS Institute, Cary, NC, USA). Differences in demographic characteristics, anthropometric variables, and scientific physical fitness measurements between BMI categories were analyzed using one-way analysis of variance (ANOVA) or chi-square tests. When a significant F value was found (*p* < 0.05), Tukey’s post hoc test was performed to determine the differences between the pairs of means. Multiple linear regression analysis with scientific physical fitness measurements as the dependent variable was used to examine the associations between scientific physical fitness measurements and BMI after adjustment for potential confounders such as age, education, occupation, monthly income, marital status, and relationship status. To examine the dose–response relationship of scientific physical fitness performance with BMI and obesity status, four different categories (quartiles) were applied to each scientific physical fitness measurement according to gender. The low quartile comprised participants who demonstrated the best performances for each physical fitness measurement, and this was assigned as the reference group for further analysis. Unconditional logistic regression analyses were conducted to evaluate the linear association between cardiorespiratory fitness, muscle fitness, or flexibility, and obesity risks. All the regression models were adjusted for age, education, occupation, monthly income, marital status, relationship status, and other scientific physical fitness measurements. Then, the adjusted odds ratios (ORs) with 95% confidence intervals (CIs) were calculated. All the data are expressed as the means ± standard deviation (SD) or frequency (percentage). The significance level adopted to reject the null hypothesis was *p* < 0.05.

## 3. Results

### 3.1. Demographic Characteristics of the Study Participants

In total, 7761 men and 9178 women were included in this study. Participants aged 23 to 64 years with complete data were included from the TSPFTP. Table 1 lists their demographic characteristics according to obesity status among adults in Taiwan. The highest proportion of participants were of a normal weight (56%). All participants were divided into a normal body group, an overweight group, and an obese group, by sex. There were significant differences between the normal weight, overweight, and obesity groups on all related variables except height for men (*p* = 0.221). For women, there were significant differences between the normal weight, overweight, and obesity groups in all related variables except relationship status (*p* = 0.236).

### 3.2. Scientific Physical Fitness Distribution according to Obesity Status

Table 2 presents the comparison of obesity status differences by various scientific physical fitness measurements including heart rate values (HR0, ∆HR3 − HR0, and ∆HR3 − HR4) among adults in Taiwan. All the obesity groups showed significant differences in all the scientific physical fitness measurements (3MPKS, grip strength/BW, and sit-and-reach tests), and the obesity group had the lowest scores for all the measurements in both men and women. However, there were no differences between the overweight and normal weight groups for either gender in the sit-and-reach test. In addition, no differences were observed between the female overweight and obesity groups in the 3MPKS test.

### 3.3. Associations of Scientific Physical Fitness Measurements with BMI

Table 3 presents the regression coefficients for predicting BMI using the different scientific physical fitness measurements. In men, adjusted for age, education, occupation, monthly income, marital status, relationship status, and other physical fitness measurements, the power was lower for the 3MPKS test (*β* = −0.127) and sit-and-reach test (*β* = −9.421) and increased for grip strength (*β* = −0.002). However, for women, after adjusting for several variables, the power was lower for both the 3MPKS test (*β* = −0.127) and grip strength (*β* = −9.421).

Table 4 presents the results of the regression coefficients for predicting BMI using different quartiles of scientific physical fitness measurements. The results indicated that there was a significant relationship between BMI and physical fitness (*p* < 0.05). In men, adjusted for age, education, occupation, monthly income, marital status, relationship status, and other physical fitness measurements, the power showed a significant difference and was highest for the 3MPKS test at the third level (*β* = 1.900) and showed the greatest decrease in grip strength at the first level (*β* = −0.922). For the sit-and-reach test, the second level showed a significant decrease (*β* = 0.178). However, in women, after adjusting for several variables, the power was significantly different and had the highest increase for the 3MPKS test at the third level (*β* = 1.454) and grip strength at the first level (*β* = −1.309), and the greatest decrease for the sit-and-reach test at the first level (*β* = 0.230).

### 3.4. Associations of Scientific Physical Fitness Measurements with Overweight Risk

Table 5 presents the multivariate adjusted ORs for overweight status in relation to the quartiles of physical fitness measurements after adjustment for potential confounders. In men, after adjusting for potential confounders, all the levels of the 3MPKS test, grip strength, and sit-and-reach test were associated with the risk of overweight compared with the reference group. In the 3MPKS test, the third level (<38.26 mL/kg/min) showed the highest risk of overweight (OR = 2.117, 95% CI: 1.734–2.586) compared with the reference group. With regard to grip strength, the first level (0.50–0.58 kg) had the highest risk of overweight (OR = 0.806, 95% CI: 0.687–0.946) compared with the reference group. In the sit-and-reach test, the second level (14.50–21.00 kg) had the highest risk of overweight (OR = 0.849, 95% CI: 0.729–0.988) compared with the reference group. 

In women, after adjusting for potential confounders, all levels of the 3MPKS test and hand grip strength were associated with a risk of overweight compared with the reference group. In 3MPKS, the third level (<31.12 mL/kg/min) had the highest risk of overweight (OR = 3.036, 95% CI: 2.467–3.738) compared with the reference group. With regard to grip strength, the first level (0.50–0.58 kg) had the highest risk of overweight (OR = 0.667, 95% CI: 0.580–0.767) compared with the reference group. In the sit-and-reach test, 27.01–34.50 cm and < 20.00 cm were associated with a risk of overweight (OR = 1.192, 95% CI: 1.019–1.394; OR = 0.798, 95% CI: 0.675–0.934) compared with the reference group.

### 3.5. Associations of Scientific Physical Fitness Measurements with Obesity Risk

The results of the logistic regression models for the risk of obesity are shown in Table 6. After adjusting for potential confounders, the results showed that participants who performed the 3MPKS test and had high handgrip strength all had a risk of obesity, and the third level of the 3MPKS test (OR = 6.530, 95% CI: 5.008–8.513) and the first level of grip strength (OR = 0.528, 95% CI: 0.446–0.624) had the highest risk of obesity in men. Performance at the second level on the sit-and-reach test was associated with a higher risk of obesity (OR = 1.376, 95% CI: 1.132–1.673) than the reference group in men.

For women, the second and third levels on the 3MPKS test were associated with a risk of obesity, and the third level had a higher risk of obesity (OR = 3.238, 95% CI: 2.505–4.187) than the reference group. Participants who performed the grip strength test all had a risk of obesity, and the first level of grip strength (OR = 0.351, 95% CI: 0.296–0.417) had the highest risk of obesity in women. In the first and second levels, the 3MPKS test was associated with a risk of obesity, and the second level had a higher risk of obesity (OR = 1.429 95% CI: 1.138–1.794) than the reference group in women.

## 4. Discussion

In this study, we analyzed the relationship between scientific physical fitness and BMI and overweight/obesity risk using data from 7761 men and 9178 women. The main findings of this study were as follows: (1) higher levels of 3MPKS and relative grip strength were each associated with lower BMI, overweight, and obesity, with a dose–response relationship in both men and women; (2) sit-and-reach was partially negatively associated with BMI and was not a good predictor of overweight/obesity in adults.

A previous study found that Caucasians had a BMI that was approximately 3 kg/m^2^ higher than Asians with the same body fat percentage, age, and sex [26]. Thus, in our study, obesity was defined as a BMI greater than or equal to 27 kg/m^2^ rather than the WHO recommendation of 30 kg/m^2^. In particular, we observed that the proportions of overweight (32% vs. 19%) and obesity (21% vs. 9%) were higher in men than in women. In contrast, the global prevalence of overweight (40% vs. 39%) and obesity (15% vs. 11%) is higher in women than in men [27]. We suggest that in Taiwan, women manage their weight more efficiently than men, which may reduce their future risk of chronic diseases and enhance their quality of life.

Cardiovascular fitness is closely associated with many health outcomes, but it is impractical to assess all populations in a laboratory setting because it requires expensive metabolic equipment and the evaluation of VO2max requires time and space and can be challenging for participants. In contrast, the 3MPKS test is a simple and validated (*r* = 0.79) method for predicting VO2max in adults [12]. In our study, we found that 3MPKS performance was significantly negatively associated with BMI and overweight/obesity risk with a dose–response relationship in both sexes. Furthermore, the risk of obesity in men and women with a VO2max below 45.4 mL/kg/min and 34.2 mL/kg/min might be 3.5–6.5 times and 1.4–3.2 times higher, respectively. A similar study indicated that low VO2max levels (35.0 vs. 20.1 mL/kg/min; measured using a cycle ergometer) in adults might increase the risk of developing obesity by 2.9 times compared with high VO2max levels [28]. In addition, higher cardiorespiratory fitness (measured by a nine-minute run or walk) was associated with a lower risk of overweight/obesity in children [29]. Therefore, the American College of Sports Medicine recommends that adults engage in moderate-intensity aerobic training (e.g., walking, running, and cycling) for ≥150 min/week or vigorous-intensity aerobic training for ≥75 min/week to improve their cardiovascular fitness and weight management [7,30].

Grip strength is a good predictor of overall body strength (i.e., a sum of shoulder abductors, hip flexors, and ankle dorsiflexors) in children and young adults [12]. One study indicated that relative grip strength was negatively associated with the risk of abdominal obesity in young adults [19]. In our study, we observed that a lower relative grip strength was associated with a higher BMI and overweight/obesity risk with a dose–response relationship in both sexes. In addition, men and women with a relative grip strength lower than 0.67 and 0.51, respectively, might have an increased risk of overweight/obesity. A similar study indicated that a lower relative grip strength was associated with a higher risk of MS in young and elderly adults [15,17]. Thus, we suggest that relative grip strength is a good predicator of overweight/obesity, abdominal obesity, and MS risks in young and older adults.

In this study, we found that sit-and-reach performance was higher in the overweight group than in the obese group, but did not differ between the overweight and normal weight groups in men and women. In addition, sit-and-reach was partially negatively associated with BMI and overweight/obesity risk in both sexes. Similar studies indicated that sit-and-reach was not affected by BMI or overweight/obesity status among children and adolescents [29,31]. Therefore, we suggest that sit-and-reach is not a good predictor of BMI distribution and overweight/obesity risk.

Our study has some limitations. First, this study did not measure body fat percentage, muscle mass, and biomarkers such as total cholesterol and triglyceride concentrations. Without these measurements, it may be difficult to comprehensively elucidate the ways in which scientific physical fitness levels affect BMI distribution and obesity prevention. Second, our questionnaire did not include a survey of physical activity or chronic diseases such as hypertension diabetes and cardiovascular disease, which might have interfered with scientific physical fitness performance. Finally, this study adopted a cross-sectional study design, and therefore no cause-and-effect relationships can be guaranteed. We recommend that future studies should be conducted using a longitudinal study design to understand the cause-and-effect relationship between scientific physical fitness and overweight/obesity risk in Taiwanese adults.

## 5. Conclusions

This study demonstrated that adults with higher cardiorespiratory fitness and muscle strength had a lower BMI and overweight/obesity risk with a dose–response relationship. Men and women with a VO2max < 45 and 34 mL/kg/min or relative grip strength < 0.67 and 0.51, respectively, might have an increased risk of overweight/obesity. The sit-and-reach test was not a good predictor of BMI distribution and overweight/obesity risk in either sex.

## Figures and Tables

**Table 1:** Demographic characteristics of the study participants according to obesity status among adults in Taiwan.

Variables	Men (*N* = 7761)				Women (*N* = 9178)			
	OB (*n* = 1627)	OW (*n* = 2495)	NW (*n* = 3639)	*p*	OB (*n* = 865)	OW (*n* = 1711)	NW (*n* = 6602)	*p*
Age (years)	38.94 ± 10.26	39.07 ± 11.25	35.83 ± 11.10	<0.0001	39.78 ± 11.36	40.61 ± 11.57	37.07 ± 10.90	<0.0001
Height (cm)	171.66 ± 6.50	171.46 ± 6.25	171.75 ± 6.44	0.221	159.37 ± 6.18	159.24 ± 6.17	159.90 ± 5.66	<0.0001
Body weight (kg)	86.71 ± 8.89	74.69 ± 6.07	64.92 ± 6.13	<0.0001	74.42 ± 7.33	64.17 ± 5,45	54.18 ± 5.21	<0.0001
BMI (kg/m^2^)	29.38 ± 1.95	25.37 ± 0.86	21.99 ± 1.39	<0.0001	29.26 ± 1.67	25.27 ± 0.83	21.17 ± 1.46	<0.0001
Education (%)				<0.0001				<0.0001
Elementary school or lower	0.7	0.8	0.3		1.2	2.9	1.2	
Junior or senior school	11.4	9.5	7.6		20.3	16.9	10.1	
College or higher	87.9	89.7	92.1		78.5	80.2	88.8	
Currently employed (%)				<0.0001				0.022
Yes	92.1	91.3	88.0		83.6	83.3	85.7	
No	4.8	5.7	8.9		14.0	14.1	11.5	
Other	3.1	3.0	3.1		2.4	2.5	2.7	
Income level (%)				<0.0001				<0.0001
≤20,000 NTD	5.7	7.9	9.2		15.5	14.3	11.3	
20,001–40,000 NTD	25.4	23.2	25.1		39.9	36.6	35.8	
≥40,001 NTD	68.9	68.9	65.7		44.6	49.1	52.8	
Marital status (%)				<0.0001				0.005
Never married	38.2	35.2	48.1		38.8	36.4	40.6	
Married	57.2	58.4	46.1		53.4	57.6	53.3	
Divorced/separation/widowed	4.5	6.4	5.8		7.7	6.0	6.1	
Relationship status (%)				<0.0001				0.148
Living with someone	83.5	82.4	78.3		88.0	85.2	86.0	
Not living with someone	16.5	17.6	21.7		12.0	14.8	14.0	

Abbreviations: BMI, body mass index; NTD, new Taiwan dollar; NW, normal weight; OB, obesity; OW, overweight; UW, underweight. Values expressed as the means ± standard deviation or percentage (%). Obesity, BMI ≥ 27 kg/m^2^; overweight, 27 > BMI ≥ 24 kg/m^2^; normal weight, 24 > BMI ≥ 18.5 kg/m^2^.

**Table 2:** Scientific physical fitness measurements according to different obesity statuses among adults in Taiwan.

Variables	OB	OW	NW	*p*	Tukey’s Post hoc Test
Men (*N* = 5764)					
3MPKS (mL/kg/min)	39.56 ± 4.02	41.23 ± 4.73	43.30 ± 5.39	<0.0001	OB < OW < NW
HR0	80.68 ± 13.38	78.82 ± 12.89	77.83 ± 13.48	<0.0001	OB > OW > NW
∆HR3 − HR0	144.82 ± 24.81	142.38 ± 23.37	143.67 ± 24.35	0.005	OB > OW
∆HR3 − HR4	120.43 ± 24.87	117.48 ± 24.45	117.32 ± 24.86	<0.0001	OB > OW, NW
Grip strength/BW	0.50 ± 0.10	0.56 ± 0.11	0.63 ± 0.13	<0.0001	OB < OW < NW
Sit-and-reach test (cm)	20.61 ± 9.38	21.94 ± 9.85	21.64 ± 9.79	<0.0001	OB < OW, NW
Women (*N* = 7639)					
3MPKS (mL/kg/min)	32.37 ± 5.21	32.82 ± 4.26	35.20 ± 4.97	<0.0001	OB, OW < NW
HR0	80.89 ± 13.03	79.41 ± 11.91	78.34 ± 13.25	<0.0001	OB > OW > NW
∆HR3 − HR0	137.28 ± 34.15	141.68 ± 28.16	141.60 ± 32.26	0.001	OB < OW, NW
∆HR3 − HR4	113.37 ± 31.93	116.10 ± 27.07	115.97 ± 29.47	0.042	OB < NW
Grip strength/BW	0.37 ± 0.08	0.41 ± 0.09	0.47 ± 0.10	<0.0001	OB < OW < NW
Sit-and-reach test (cm)	26.25 ± 9.49	27.72 ± 9.70	27.51 ± 10.67	0.002	OB < OW, NW

Abbreviations: 3MPKS, 3 min progressive knee-up and step; BW, body weight; NW, normal weight; OB, obese; OW, overweight; SD, standard deviation. Values are expressed as the means ± SD. Obesity, BMI ≥ 27 kg/m^2^; overweight, 27 > BMI ≥ 24 kg/m^2^; normal weight, 24 > BMI ≥ 18.5 kg/m^2^.

**Table 3:** Regression coefficients for predicting BMI using different scientific physical fitness measurements.

Variables	Model 1 (Unadjusted)			Model 2 (Adjusted ^a^)		
	*β*	SE	*p*	*β*	SE	*p*
Men						
3MPKS (mL/kg/min)	−0.116	0.277	<0.0001	−0.127	0.008	<0.0001
Grip strength/BW	−8.970	0.264	<0.0001	−9.421	0.271	<0.0001
Sit-and-reach test (cm)	−0.005	0.003	0.120	−0.002	0.003	0.522
Women						
3MPKS (mL/kg/min)	−0.108	0.006	<0.0001	−0.111	0.007	<0.0001
Grip strength/BW	−10.227	0.284	<0.0001	−10.096	0.285	<0.0001
Sit-and-reach test (cm)	0.007	0.003	0.007	0.007	0.003	0.006

Abbreviations: 3MPKS, 3 min progressive knee-up and step; BMI, body mass index; BW, body weight; SE, standard error; *β*, regression coefficient. ^a^ Adjusted for age, education, occupation, monthly income, marital status, relationship status, and other scientific physical fitness measurements.

**Table 4:** Regression coefficients for predicting BMI using different quartiles of scientific physical fitness measurements.

Variables	Model 1 (Unadjusted)			Model 2 (Adjusted ^a^)		
	*β*	SE	*p*	*β*	SE	*p*
Men						
3MPKS (mL/kg/min)						
>45.43	Ref.	—	—	Ref.	—	—
41.62–45.43	1.078	0.092	<0.0001	1.079	0.095	<0.0001
38.26–41.61	1.524	0.094	<0.0001	1.594	0.102	<0.0001
<38.26	1.749	0.095	<0.0001	1.900	0.117	<0.0001
Test for trend	*p* < 0.0001			*p* < 0.0001		
Grip strength/BW						
<0.50	Ref.	—	—	Ref.	—	—
0.50–0.58	−0.814	0.090	<0.0001	−0.922	0.091	<0.0001
0.59–0.67	−1.758	0.093	<0.0001	−1.865	0.094	<0.0001
>0.67	−3.011	0.097	<0.0001	−3.108	0.099	<0.0001
Test for trend	*p* < 0.0001			*p* < 0.0001		
Sit-and-reach test (cm)						
>28.00	Ref.	—	—	Ref.	—	—
21.01–28.00	0.043	0.091	0.633	−0.004	0.091	0.961
14.50–21.00	0.227	0.090	0.012	0.178	0.090	0.048
<14.50	−0.024	0.092	0.795	−0.082	0.092	0.371
Test for trend	*p* = 0.576			*p* = 0.888		
Women						
3MPKS (mL/kg/min)						
>37.59	Ref.	—	—	Ref.	—	—
34.24–37.59	0.528	0.080	<0.0001	0.555	0.081	<0.0001
31.12–34.23	0.864	0.081	<0.0001	0.882	0.085	<0.0001
<31.12	1.484	0.082	<0.0001	1.454	0.096	<0.0001
Test for trend	*p* < 0.0001			*p* < 0.0001		
Grip strength/BW						
<0.38	Ref.	—	—	Ref.	—	—
0.38–0.45	−1.349	0.078	<0.0001	−1.309	0.079	<0.0001
0.46–0.51	−2.206	0.085	<0.0001	−2.161	0.085	<0.0001
>0.51	−2.810	0.084	<0.0001	−2.759	0.085	<0.0001
Test for trend	*p* < 0.0001			*p* < 0.0001		
Sit-and-reach test (cm)						
>34.50	Ref.	—	—	Ref.	—	—
27.01–34.50	0.231	0.080	0.004	0.230	0.080	0.004
20.00–27.00	0.207	0.080	0.010	0.209	0.080	0.009
<20.00	−0.186	0.080	0.020	−0.185	0.080	0.021
Test for trend	*p* = 0.022			*p* = 0.025		

Abbreviations: 3MPKS, 3 min progressive knee-up and step; BMI, body mass index; BW, body weight; SE, standard error; *β*, regression coefficient. ^a^ Adjusted for age, education, occupation, monthly income, marital status, relationship status, and other scientific physical fitness measurements.

**Table 5:** Multivariate adjusted ORs for overweight in relation to quartiles of scientific physical fitness measurements after adjustment for potential confounders (n = 14,447).

Variables	Model 1 (Unadjusted)			Model 2 (Adjusted ^a^)		
	OR	95% CI	*p*	OR	95% CI	*p*
Men						
3MPKS (mL/kg/min)						
>45.43	Ref.	—	—	Ref.	—	—
41.62–45.43	2.272	1.955–2.641	<0.0001	2.063	1.766–2.410	<0.0001
38.26–41.61	2.423	2.074–2.832	<0.0001	2.056	1.731–2.442	< 0.0001
<38.26	2.655	2.263–3.115	<0.0001	2.117	1.734–2.586	<0.0001
Test for trend	*p* < 0.0001			*p* < 0.0001		
Grip strength/BW						
<0.50	Ref.	—	—	Ref.	—	—
0.50–0.58	0.850	0.727–0.994	0.042	0.806	0.687–0.946	0.008
0.59–0.67	0.750	0.643–0.874	<0.0001	0.697	0.595–0.817	<0.0001
>0.67	0.305	0.258–0.361	<0.0001	0.278	0.234–0.330	<0.0001
Test for trend	*p* < 0.0001			*p* < 0.0001		
Sit-and-reach test (cm)						
>28.00	Ref.	—	—	Ref.	—	—
21.01–28.00	0.843	0.725–0.980	0.026	0.810	0.696–0.942	0.006
14.50–21.00	0.872	0.750–1.014	0.074	0.849	0.729–0.988	0.035
<14.50	0.816	0.701–0.950	0.009	0.781	0.670–0.911	0.002
Test for trend	*p* = 0.074			*p* = 0.015		
Women						
3MPKS (mL/kg/min)						
>37.59	Ref.	—	—	Ref.	—	—
34.24–37.59	1.930	1.605–2.320	<0.0001	1.959	1.625–2.361	<0.0001
31.12–34.23	2.562	2.141–3.066	<0.0001	2.542	2.107–3.067	<0.0001
<31.12	3.299	2.759–3.945	<0.0001	3.036	2.467–3.738	<0.0001
Test for trend	*p* < 0.0001			*p* < 0.0001		
Grip strength/BW						
<0.38	Ref.	—	—	Ref.	—	—
0.38–0.45	0.645	0.561–0.741	<0.0001	0.667	0.580–0.767	<0.0001
0.46–0.51	0.384	0.326–0.451	<0.0001	0.398	0.338–0.469	<0.0001
>0.51	0.218	0.182–0.262	<0.0001	0.228	0.190–0.274	<0.0001
Test for trend	*p* < 0.0001			*p* < 0.0001		
Sit-and-reach test (cm)						
>34.50	Ref.	—	—	Ref.	—	—
27.01–34.50	1.195	1.022–1.397	0.025	1.192	1.019–1.394	0.028
20.00–27.00	1.080	0.922–1.263	0.340	1.085	0.927–1.272	0.310
<20.00	0.787	0.669–0.925	0.004	0.794	0.675–0.934	0.005
Test for trend	*p* = 0.002			*p* = 0.003		

Abbreviations: 3MPKS, 3 min progressive knee-up and step; BW, body weight; CI, confidence interval; OR, odds ratio. ^a^ Adjusted for age, education, occupation, monthly income, marital status, relationship status, and other scientific physical fitness measurements.

**Table 6:** Multivariate adjusted ORs for obesity in relation to quartiles of scientific physical fitness measurements after adjustment for potential confounders (n = 12,733).

Variables	Model 1 (Unadjusted)			Model 2 (Adjusted ^a^)		
	OR	95% CI	*p*	OR	95% CI	*p*
Men						
3MPKS (mL/kg/min)						
>45.43	Ref.	—	—	Ref.	—	—
41.62–45.43	3.488	2.757–4.412	<0.0001	3.557	2.798–4.522	<0.0001
38.26–41.61	5.375	4.277–6.757	<0.0001	5.747	4.509–7.326	<0.0001
<38.26	5.760	4.580–7.243	<0.0001	6.530	5.008–8.513	<0.0001
Test for trend	*p* < 0.0001			*p* < 0.0001		
Grip strength/BW						
<0.50	Ref.	—	—	Ref.	—	—
0.50–0.58	0.593	0.504–0.697	<0.0001	0.528	0.446–0.624	<0.0001
0.59–0.67	0.237	0.197–0.286	<0.0001	0.206	0.169–0.250	<0.0001
>0.67	0.056	0.043–0.073	<0.0001	0.049	0.037–0.064	<0.0001
Test for trend	*p* < 0.0001			*p* < 0.0001		
Sit-and-reach test (cm)						
>28.00	Ref.	—	—	Ref.	—	—
21.01–28.00	1.054	0.865–1.284	0.602	0.981	0.803–1.198	0.847
14.50–21.00	1.443	1.191–1.748	<0.0001	1.376	1.132–1.673	0.001
<14.50	0.983	0.809–1.195	0.865	0.900	0.737–1.099	0.303
Test for trend	*p* = 0.405			*p* = 0.986		
Women						
3MPKS (mL/kg/min)						
>37.59	Ref.	—	—	Ref.	—	—
34.24–37.59	0.900	0.696–1.163	0.419	0.958	0.739–1.242	0.745
31.12–34.23	1.289	1.016–1.635	0.037	1.431	1.116–1.833	0.005
<31.12	2.633	2.116–3.277	<0.0001	3.238	2.505–4.187	<0.0001
Test for trend	*p* < 0.0001			*p* < 0.0001		
Grip strength/BW						
<0.38	Ref.	—	—	Ref.	—	—
0.38–0.45	0.344	0.290–0.408	<0.0001	0.351	0.296–0.417	<0.0001
0.46–0.51	0.126	0.098–0.161	<0.0001	0.129	0.100–0.166	<0.0001
>0.51	0.052	0.037–0.073	<0.0001	0.051	0.036–0.072	<0.0001
Test for trend	*p* < 0.0001			*p* < 0.0001*		
Sit-and-reach test (cm)						
>34.50	Ref.	—	—	Ref.	—	—
27.01–34.50	1.408	1.119–1.773	0.004	1.396	1.108–1.758	0.005
20.00–27.00	1.470	1.172–1.844	0.001	1.429	1.138–1.794	0.002
<20.00	1.061	0.842–1.337	0.614	1.035	0.820–1.305	0.774
Test for trend	*p* = 0.950			*p* = 0.868		

Abbreviations: 3MPKS, 3 min progressive knee-up and step; BW, body weight; CI, confidence interval; OR, odds ratio. ^a^ Adjusted for age, education, occupation, monthly income, marital status, relationship status, and other scientific physical fitness measurements.

## Data Availability

The data that support the findings of this study are available from [the Sports Cloud: Information and Application Research Center of Sports for All, Sport Administration, Ministry of Education in Taiwan], but restrictions apply to the availability of these data, which were used under license for this study and are not publicly available. Data are, however, available from the authors upon reasonable request and with the permission of [the Sports Cloud: Information and Application Research Center of Sports for All, Sport Administration, Ministry of Education in Taiwan].
